# Are All Retinal Nerve Fiber Layer Defects on Optic Coherence Tomography Glaucomatous?

**DOI:** 10.4274/tjo.86461

**Published:** 2017-10-27

**Authors:** Sirel Gür Güngör, Akman Ahmet

**Affiliations:** 1 Başkent University Faculty of Medicine Department of Ophthalmolgy, Ankara, Turkey

**Keywords:** Anterior ischemic optic neuropathy, glaucoma, optic coherence tomography, Retinal nerve fiber layer

## Abstract

**Objectives:**

In this study, we investigated the patients who were referred to our clinic with a prediagnosis of glaucoma based on retinal nerve fiber layer (RNFL) defects on optic coherence tomography (OCT) but were determined to have nonglaucomatous RNLF defects upon detailed examination.

**Materials and Methods:**

The ophthalmic examination notes, OCT images, Heidelberg retinal tomography (HRT) II and fundus photographs of 357 patients were retrospectively evaluated. Final diagnoses of these patients were investigated.

**Results:**

Of the 357 patients, 216 (60.5%) were diagnosed as open angle glaucoma, 33 (9.2%) as low-tension glaucoma, 39 (10.9%) as pre-perimetric glaucoma. The ophthalmic examinations of 14 patients (3.9%) were normal and there were no RNFL defects in OCT examinations after dilatation. In 39 patients (10.9%), the ophthalmic and optic disc examinations were completely normal and no etiologic factor explaining RNFL defects was found. Twenty-two eyes of 16 patients (4.5%) were included in this study (the mean age was 53.8±11.5 years; 9 men and 7 women). After detailed questioning of the medical history and systemic and neurologic examinations, a diagnosis of ischemic optic neuropathy was made in 11 eyes (10 patients) (2.8%), optic neuritis in 3 eyes (2 patients) (0.6%), optic disc drusen in 4 eyes (2 patients) (0.6%), pseudotumor cerebri in 2 eyes (1 patient) (0.3%), and cerebral palsy in 2 eyes (1 patient) (0.3%).

**Conclusion:**

Decrease in RNFL thickness on OCT images alone may be misleading in glaucoma examination. In cases where optic disc cupping is not evident, diagnosis should not be based on OCT RNFL examinations alone, and the patient’s medical history, detailed ophthalmic examination, OCT optic disc parameters, HRT, and visual field tests should all be carefully evaluated together.

## INTRODUCTION

Optical coherence tomography (OCT) was first used in the 1990s for glaucoma and retinal diseases.^1,2,3,4^ Today, it is increasingly used in nearly all sub-specialties of ophthalmology.

Glaucomatous optic neuropathy is characterized by thinning of the peripapillary retinal nerve fiber layer (RNFL) and optic disc cupping as a result of axonal and secondary retinal ganglion cell loss. RNFL defects on OCT are one of the earliest signs of glaucoma.^5,6,7,8,9^ In ophthalmology practice, clinicians sometimes have difficulty with the differential diagnosis of RNFL defects resembling glaucoma, but it may be possible to distinguish these cases from glaucomatous eyes with a careful fundus examination and Heidelberg retinal tomography (HRT) II imaging.^10^

RNFL thinning is not specific to glaucomatous optic neuropathy and may also be seen in various nonglaucomatous optic neuropathies and central nervous system diseases.^11,12,13,14^ In such clinical cases, RNFL thinning accompanied by optic disc cupping may be considered a finding in favor of glaucoma.^6,7,8,9,10^

In this study, we reviewed the examination findings and etiologies of patients who were referred to our glaucoma outpatient clinic with possible glaucomatous RNFL damage but were found to have nonglaucomatous RNFL defects upon detailed ophthalmologic examination, OCT, and HRT II evaluation.

## MATERIALS AND METHODS

The names of 357 patients referred to our clinic for suspected or prediagnosed glaucoma based on RNFL defect identified during ophthalmologic examination and OCT between 2011 and 2015 were noted. The study was approved by Başkent University Institutional Review Board (project no: KA17-240) and supported by Başkent University Research Fund. Data regarding detailed ophthalmologic and systemic history, best corrected visual acuity, intraocular pressure measurement by applanation tonometry, central corneal thickness, and slit-lamp anterior segment and dilated fundus examinations were recorded. The patients had also undergone fundus photography and 24-2 visual field, OCT, and HRT II imaging. Patients with ophthalmologic diseases other than neuroophthalmologic conditions that may affect visual field, OCT and HRT II, those with spherical refraction greater than ±5 diopter (D) or astigmatism greater than ±3 D, those who had previous ocular surgery other than cataract surgery, those whose HRT II images were of poor quality, and those who had less than 5/10 signal strength on OCT were excluded from the study.

Non-arteritic ischemic optic neuropathy (NAION) diagnosis was based on a history of acute, painless incomplete vision loss combined with optic disc edema, and superficial hemorrhage at the margins of the optic disc in the adjacent retinal area.^15,16,17^ The patients were asked in detail about signs and symptoms suggestive of arteritic anterior ischemic optic neuropathy as described by Beck et al.^18^ These include systemic symptoms of giant cell arteritis, sudden decrease in visual acuity to hand movement or lower, chalky white edema of the optic disc, occlusion of one or more posterior ciliary arteries in fundus fluorescein angiography, and high erythrocyte sedimentation rate (55 mm/hour).

Visual field was assessed via 24-2 full-threshold test using Humphrey automatic perimetry (Humphrey Instruments, Inc., Dublin, California, USA). The test was repeated for patients with over 33% false positivity and false negativity and over 20% fixation loss. Cirrus HD spectral domain OCT (Carl Zeiss Meditec, Dublin, CA, USA) was used to evaluate the optic disc head and RNFL. For HRT II (HRT II, Heidelberg Engineering, Dossenheim, Germany) measurements, three topographical images were obtained from each patient and automatically rendered into a single averaged topographical image for analysis. An experienced technician identified the optic disc margins on the averaged topographical image using a color photograph of the optic disc.

Glaucoma diagnosis was based on a lack of optic disc pallor in patients having visual field defect consistent with typical glaucomatous optic disc appearance. Daytime measurements were repeated for patients whose intraocular pressure measurements were below 21 mmHg. Typical glaucomatous optic disc appearance involves vertical cup-to-disc (C/D) ratio greater than 0.6, localized thinning of the neural rim, splinter hemorrhages, and/or visible nerve fiber layer defect.

Patients were noted as having nonglaucomatous RNFL damage when RNFL defect was detected on OCT but the optic disc appeared nonglaucomatous in both dilated fundus examination and optic nerve topographic imaging by OCT and HRT (C/D ratio <0.6, optic disc parameters within normal limits). These patients’ ophthalmologic data and results of neurologic consultation were analyzed retrospectively.

## RESULTS

Of the 357 patients referred to our clinic for RNFL thinning, 216 (60.5%) were diagnosed with open-angle glaucoma, 33 (9.2%) with low-pressure glaucoma, and 39 (10.9%) with pre-perimetric glaucoma. In 14 patients (3.9%), ophthalmologic examination was normal and no RNFL damage was detected in OCT examination. In 39 patients (10.9%), ophthalmologic and optic disc examinations were completely normal. OCT and HRT disc parameters were appropriate for the patients’ ages. No etiological factors were found to explain RNFL defect on OCT. These patients were followed for approximately 25.2±12.31 months (12-47 months) and exhibited no progression. The RNFL damage seen in these patients was considered artifact or anatomic variation.

Twenty-two eyes of 16 patients (4.5%) were included in our analysis. Optic disc examination of these eyes revealed no glaucomatous cupping. Both OCT and HRT II disc topographic analyses were consistent with the optic disc examinations, and the C/D ratios were normal. The mean age of these patients was 53.8±11.5 years (43-70 years). Nine were male, 7 were female. The average corrected visual acuity of the patients was 0.8±0.4 (0.05-1.0). All of the patients had intraocular pressure values below 21 mmHg. The average intraocular pressure was 16.2±12.3 mmHg (8-19 mmHg) and the average central corneal thickness was 546.32±24.46 µm (536-587 µm). Eighteen eyes of 14 patients exhibited RNFL damage with optic disc pallor. The disc parameters determined by OCT and HRT are presented in Tables 1 and 2.

OCT revealed superior, temporal, and inferior RNFL damage in 8 eyes; superior, nasal, and inferior RNFL damage in 4 eyes; superior and temporal RNFL damage in 4 eyes; and 360o RNFL damage in 6 eyes. Table 3 shows the distribution of RNFL defects based on disease.

Nine eyes had generalized depression of the visual field, 2 had superotemporal quadrantanopsia, 1 had superior and inferior arcuate defect, 1 had depression in the nasal quadrant, and 5 had inferior hemifield defect. There was no visual field loss in 4 eyes. Patients with no visual field loss had RNFL defects associated with optic disc drusen. Evaluation of visual field parameters revealed a median deviation of -11.7±10.8 (-31.12-0.62) and pattern standard deviation of 6.5±4.2 (1.6-13.05).

**Figure 1:**
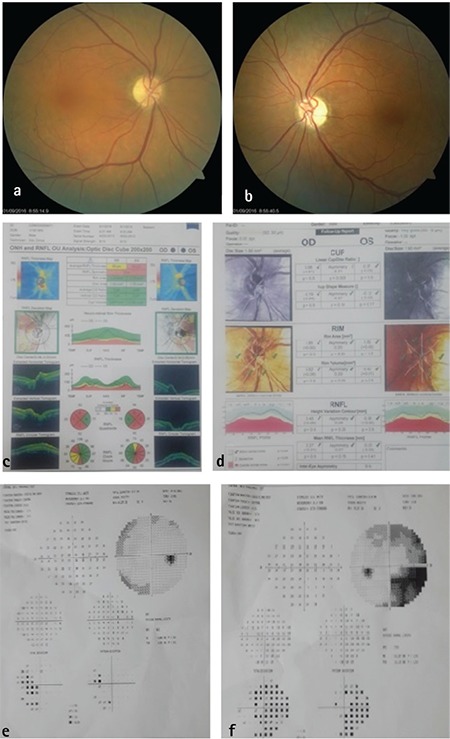
A 65-year-old male patient with bilateral non-arteritic ischemic optic neuropathy: a, b) There is no cupping in either eye and optic disc pallor is evident in the left eye; c) Optical coherence tomography shows retinal nerve fiber layer (RNFL) defects in both eyes; d) Heidelberg retinal tomography images are within normal limits; e, f) A visual field defect is detected in the region corresonding to RNFL thinning

**Figure 2:**
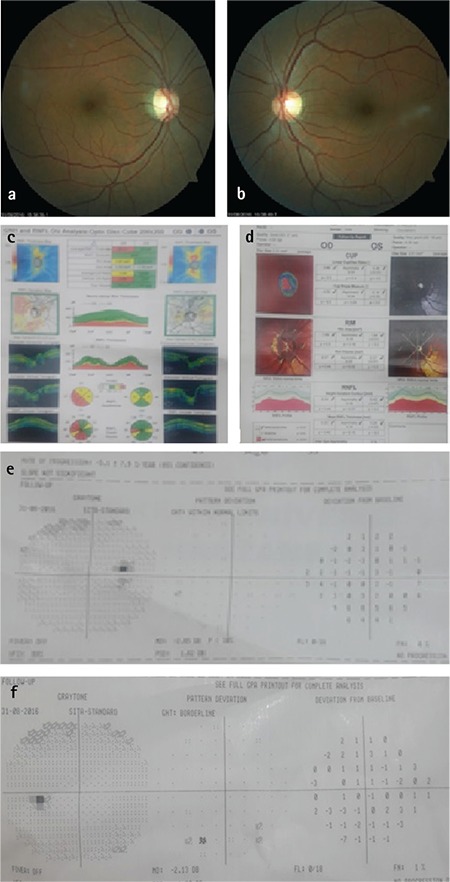
A patient with history of multiple sclerosis-associated retrobulbar optic neuritis. Optic disc examination, visual field test, and Heidelberg retinal tomography are within normal limits, while optical coherence tomography reveals retinal nerve fiber layer defect in the right eye

Upon detailed review of these patients’ medical history and systemic and neurological examination, 11 eyes (10 patients) (2.8%) were diagnosed with previous ischemic optic neuropathy (Figure 1), 3 eyes (2 patients) (0.6%) with optic neuritis associated with multiple sclerosis (MS) (Figure 2), 4 eyes (2 patients) (0.6%) with optic disc drusen, 2 eyes (1 patient) (0.3%) with pseudotumor cerebri (PTC), and 2 eyes (1 patient) (0.3%) with cerebral palsy. The distribution of etiological factors according to the RNFL defects is shown in Table 4. Ischemic optic neuropathy, MS, PTC, and cerebral palsy are presented collectively as neuroophthalmologic diseases.

Of the patients diagnosed with NAION (10 patients, 11 eyes), 3 were followed in our clinic in the acute period, but the remaining 7 were diagnosed after reviewing the symptoms with the patient and requesting a detailed examination report from the hospital where the patient was treated. For all patients, at least 6 months had passed since the acute optic disc edema period and the optic disc margins could be clearly distinguished during the study.

## DISCUSSION

RNFL thinning is seen on OCT in both glaucoma and nonglaucomatous optic neuropathies and central nervous system diseases.^12,19,20^ These diseases include ischemic optic neuropathy, optic neuritis, hereditary optic neuropathy, traumatic optic neuropathy, MS, and degenerative diseases such as Alzheimer’s and Parkinson’s disease.^11,12,13,14,19,20,21,22^

In our study, NAION was the most common diagnosis in patients with RNFL thinning who presented with suspected glaucoma. NAION is believed to be caused by acute perfusion deficiency around the optic nerve head. Although the actual etiology has not yet been clarified, a possible reason is reduced circulation to the posterior ciliary arteries.^23,24^ NAION patients have small disc area, no or minimal physiological cupping, and characteristic disc structures such as “crowded disc”, in which there is an excessive number of central retinal vein branches within the disc.^25^ Horowitz et al.^13^ compared RNFL thickness measured by OCT (at least 6 months after loss of vision) of 18 AION eyes with hemifield defect with the RNFL thickness of 29 glaucomatous eyes with hemifield defect, and found that the RNFL in the area corresponding to the visual field defect was similar between the two groups. However, the authors reported that the glaucomatous eyes exhibited more extensive RNFL thinning in quadrants which were unrelated to the visual field loss. They attributed this difference to the etiological differences underlying the RNFL thinning in glaucoma and NAION.

The acute phase of NAION does not mimic glaucoma symptoms. The optic nerve edema occurring in acute NAION is associated with increased RNFL thickness; Contreras et al.^26^ showed that RNFL thickness in eyes with acute NAION was 96.4% greater than in the patients’ fellow eyes. At 6-month follow-up, the RNFL of these patients was thinnest in the superior quadrant, followed by inferior, temporal, and nasal quadrants. This pattern explains why the inferior visual field is most affected in eyes with AION.^27^

Yang et al.^28^ used Fourier domain OCT to compare optic nerve head and RNFL thickness in individuals with glaucoma, with NAION, and in healthy individuals. In their study, glaucomatous eyes exhibited larger cup area and volume, higher C/D ratio, and smaller rim area and disc volume. NAION eyes had the smallest cup area and volume, while their rim area and volume and disc volume were comparable to those of the control group. While RNFL thickness is most commonly seen in the superotemporal and inferotemporal zones in glaucomatous eyes, it is mostly seen in the superonasal region in eyes with NAION. In our study, eyes with NAION exhibited RNFL thinning in the superonasal and inferior zones in 2 eyes, whereas the superotemporal RNFL was affected in 1 eye, the superotemporal and inferior RNFL in 6 eyes, and 360° RNFL in 2 eyes.

MS patients may have subclinical RNFL thinning, even if they have no history of optic neuritis.^29,30,31^ The etiology of RNFL damage in 3 eyes of 2 patients included in this research was attributed to MS and previous optic neuritis history. Bock et al.^30^ compared RNFL thickness measurements in OCT of patients with and without optic neuritis history and patients with glaucomatous optic neuropathy. They found that although the average and quadrant RNFL thicknesses in all three groups were less than those of the control group, there were no differences in RNFL thickness between the three groups. Temporal peripapillary area was found to be thinner in patients with a history of optic neuritis. The patients included in our study also exhibited similar thinning in the temporal RNFL. In a study conducted by Yılmazbaş et al.^32^, the RNFL thickness of affected eyes in patients with MS-associated unilateral optic neuritis was lower when compared to their healthy fellow eyes and to the control group. Daldal et al.^33^ compared the RNFL thickness of patients with MS-related optic neuritis history and patients with no optic neuritis history to a healthy control group, and found a greater reduction in RNFL thickness in the eyes with history of optic neuritis. In their study, RNFL thinning occurred most commonly in the temporal zone, as in our research.

In our study, we identified optic disc drusen in 4 eyes of 2 patients during glaucoma examination performed due to RNFL loss. Optic disc drusen may damage nerve axons, consequently leading to RNFL defects. These defects may affect the visual field and raise suspicion of glaucoma.^34^ In addition, drusen may apply local pressure to the venous and arterial vessels, resulting in thinning of the RNFL. There have been few studies on the differential diagnosis between glaucoma and RNFL defects caused by drusen. Roh et al.^35^ demonstrated that patients with optic disc drusen have thinner RNFL in the superior and inferior quadrants compared to a healthy control group. Of the 4 eyes with optic disc drusen that were included in our study, 2 had superior, nasal, and inferior RNFL thinning and 2 had superior and temporal RNFL thinning. None of the patients with optic disc drusen included in our study had visual field defect due to RNFL thinning. In patients with optic disc drusen who develop glaucoma, it may be difficult to identify the exact cause of RNFL damage and prescribe treatment. In such cases, it is useful to monitor RNFL damage with progression analysis methods. However, it should be kept in mind that RNFL damage may continue in both conditions.

PTC was identified in the detailed examination of a patient with RNFL defect that was included in our study. The patient had been prescribed oral acetazolamide treatment but did not regularly attend follow-up appointments. Chronic papilledema is a major cause of progressive and permanent vision loss in PTC patients.^36,37,38^ RNFL thickness increases during the papilledema period and OCT may be used to monitor optic disc edema. There are studies in the literature reporting improvements in RNFL thickness and visual field parameters after PTC treatment.^39,40^ Other studies have also suggested that OCT can be used to monitor the results of medical and surgical treatment of PTC.^41,42^ However, in the presence of papilledema it is not possible to quantify retinal nerve loss using OCT because RNFL thickness increases in optic disc edema, thus preventing the detection of axonal loss.^43^ When patients develop optic atrophy in the chronic period, RNFL thinning is already an expected outcome.^44^

In the present study, one patient was diagnosed with cerebral palsy. This patient’s optic disc did not appear glaucomatous and there was optic atrophy. Cerebral palsy may be accompanied by unilateral or bilateral optic atrophy. The most common cause of optic atrophy in cerebral palsy is direct trauma to the orbit during birth or, less frequently, trauma to the base of the skull.^45,46,47^

## CONCLUSION

RNFL thinning may occur in neuroophthalmologic diseases and optic disc anomalies. In the differential diagnosis of glaucomatous and nonglaucomatous optic neuropathies, OCT evaluation should include not only RNFL measurements but also disc topography parameters. A careful ophthalmologic examination and HRT optic disc topography, if available, can also facilitate differential diagnosis.

## Figures and Tables

**Table 1:**
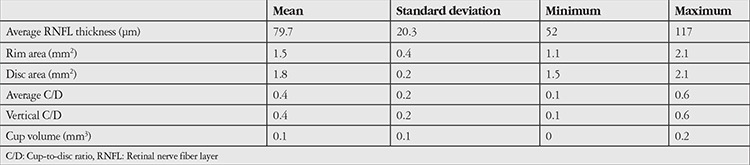
Optic disc parameters on optical coherence tomography in patients with nonglaucomatous retinal nerve fiber layer damage

**Table 2:**
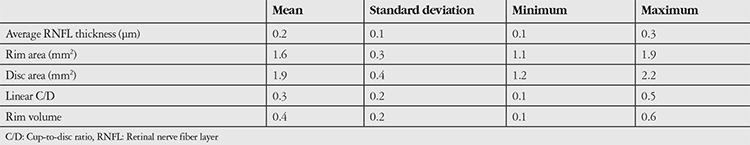
Optic disc parameters on Heidelberg retinal tomography in patients with nonglaucomatous retinal nerve fiber layer damage

**Table 3:**
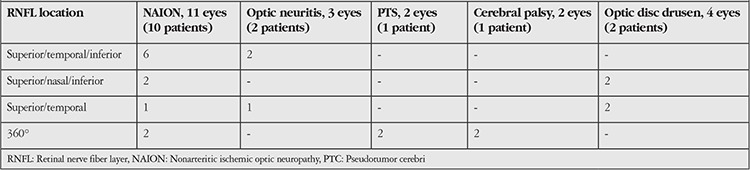
Distribution of retinal nerve fiber layer damage according to disease

**Table 4:**
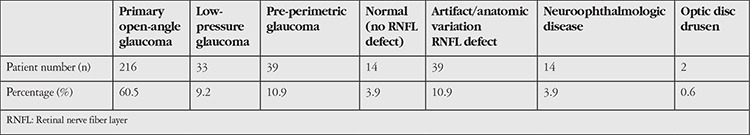
Prevalence of retinal nerve fiber layer defects according to etiology
